# Educational neuroscience, constructivism, and the mediation of learning and creativity in the 21st century

**DOI:** 10.3389/fpsyg.2015.00133

**Published:** 2015-02-12

**Authors:** M. Layne Kalbfleisch

**Affiliations:** ^1^Kidlab, Krasnow Institute, College of Education and Human Development, George Mason UniversityFairfax, VA, USA; ^2^Pediatrics, The George Washington University School of Medicine and Health SciencesWashington, DC, USA

**Keywords:** educational neuroscience, transfer, neuroplasticity, reasoning, neuromyth, creativity, math, music

Elephant. Imagine one standing right in front of you. Why begin this editorial on educational neuroscience and constructivist learning with an elephant? Because in many ways, this book illustrates the old parable of the blind man who has his hand on the elephant. More specifically, he has his hand on *part* of the elephant (his flank? his trunk?). And that part, in and of itself, informs the blind man's understanding of the elephant as the thing in itself. Yet, a compadre standing with him also has *his* hand on the elephant and commands his own subjective conception of and experience with the great mammal.

The call for this special topic was intentionally broad and conceptually ambitious, to coalesce state-of-the art research across multiple domains that would collectively give shape to the emerging field of educational neuroscience. This book spans a body of work that represents the efforts of 60 contributors and took 14 months to publish from the first article (on neuromyth) to the last (on the multiple intelligences). That shape, provided by nine original research papers, two reviews, and one perspective article, pragmatically demonstrates one of the most difficult existing theoretical proofs in the field of educational psychology—constructivist learning. Both ubiquitous in its influence (the grand challenge?) and hard to parse (the elephant in the room?), each paper in this book gives rise to a piece of the elephant *constructivism*. An idea blueprinted by Vygotsky, Dewey, and Bruner, and now instantiated in this series of papers, tackles the issues of learning, transfer, and experience with multiple metrics (from survey research to various neuroimaging techniques) across domain general (creativity, reasoning) and specific (“three R” learning, music) processes and accounts for the influences of state (motivation), genetics (in reading disability), and arousal (sleepiness). Three aspects of constructivism arise from this collective: the influence of context on human learning and performance, training influences on neural plasticity and learning transfer, and the assessment of individual differences. This modern interpretation illustrates the potential of educational neuroscience to answer “how,” “why,” and “when” individual differences matter and when we've struck upon a piece of universal knowledge.

Intervention science is still in its infancy and overlaps our efforts to norm the basic developmental trajectories of the brain across life. In my opinion, learning context is the great divide between educators and neuroscientists simply because the scales of measurement are so different. In response, these papers are examples of the intellectual risk, curiosity, and transdisciplinary thought that narrow that divide. For example, musicologist Alexander Khalil poses the idea that training young children to synchronize their playing of the Balinese gamelan may, perhaps, influence more general tendencies to self-regulate and attend. In that same vein, Nina Kraus' team reveals that music training in adolescence may help the brain better contend with distraction. Todd Lubart and his team add “matter” into the ongoing discussion about the contingencies of creativity, what it is and means in different contexts, and how we might assess it usefully. Jelle Jolles, Paul Howard Jones and colleagues attempt an “epidemiology” of neuromyth in order to assess the influence of this roadblock in our understanding of the learning process. Other articles span a range that includes: articulating rules and methods for understanding the relationship between genetics and behavior in reading disability, assessing how sleep quality may impact performance in school, the context in which transfer may occur between linguistic training, reading, and writing skills in college students, and arithmetic fact learning and retrieval in numerical and nonverbal problem solving contexts.

In an age where complexity is beginning to define how we understand our cities, other pandemic dynamical systems, and even ourselves, we cannot, for the sake of education, afford to dismiss this theory's complexity. Because it is articulated from multiple historical and contemporary perspectives suggests an explanatory power that, while complex, will eventually simplify and collapse our understanding of the learning process to the best set of first principles that can re-populate and position our understanding of what education ought to be and look like no matter the context. After all, that is the goal of educational neuroscience—to apply the tools, metrics, and methods of neuroscience to questions and problems of human learning in order to inform aspects of curriculum design, pedagogy, and human performance in both formal and informal learning contexts.

Though this research topic is conceptually broad and perhaps “wider than the sky” would say Emily Dickinson, it is a necessary first cut to re-define the blueprint of constructivist learning according to the tools, metrics, and methods that modern neuroscience provides us. And with that, the need to educate readers so that they become literate consumers of this new technical genre so that we can arrest the impact of existing neuromythologies and curtail the proliferation of new ones. And so the elephant emerges again as a metaphor. Elephants possess a fantastic memory. They remind us how fundamental the learning process is to survival and a capacity to thrive individually and socially. They remind us that though the proposition of understanding learning is impossibly large, that we can take a crack at it, see what happens when we do, and, in the end, through new knowledge and reflection, learn from our own experience.

## Conflict of interest statement

The author declares that the research was conducted in the absence of any commercial or financial relationships that could be construed as a potential conflict of interest.

